# Total Knee Replacement Among Elderly: The Role of Tranexamic Acid

**DOI:** 10.7759/cureus.71443

**Published:** 2024-10-14

**Authors:** Abdullah Almelaifi, Mohammed K Alghamdi, Alwaleed A Alqarni, Ali M Al Ajmi, Abdulrahman A ALShehri, Saad N Al-Harthi, Naif M Alhamam

**Affiliations:** 1 College of Medicine and Surgery, King Faisal University, Al Khobar, SAU; 2 Medicine, King Faisal University, Al Khobar, SAU; 3 Medicine, King Faisal University, Dammam, SAU; 4 Medicine, King Faisal University, Al Hofuf, SAU; 5 Ophthalmology, King Faisal University, Al Hofuf, SAU; 6 College of Medicine, King Faisal University, Al Hofuf, SAU; 7 Orthopedics, King Faisal University, Al Hofuf, SAU

**Keywords:** antifibrinolytic agent, postoperative blood loss, total knee arthroplasty, total knee replacement (tkr), tranexamic acid (txa)

## Abstract

Background

Tranexamic acid (TXA) administration in total knee replacement (TKR) surgery has been shown to reduce blood loss and transfusion requirements. However, its efficacy and safety in elderly patients undergoing arthroplasty remain under investigation. This study aimed to assess the impact of TXA on blood loss and post-operative outcomes in TKR patients among a local elderly population.

Methodology

A prospective descriptive-analytical hospital-based randomized study was conducted, involving 79 TKR patients who received TXA. Demographic data, comorbidities, pre- and post-operative hemoglobin levels, length of hospital stay, transfusion requirements, and post-operative complications were recorded. Statistical analysis was performed using SPSS (IBM Corp., Armonk, NY), including descriptive analysis and comparative tests.

Results

The mean drop in hemoglobin levels post-operatively was -1.55 g/dL (SD = 0.94). The majority of patients (78, 98.7%) did not require packed red blood cell transfusions post-operatively, and complications were minimal (1, 1.3%). The length of hospital stay was relatively short (mean = 4.84 days). Comparison with existing literature revealed TXA's effectiveness in reducing blood loss compared to studies without TXA administration.

Conclusion

TXA administration in TKR surgery effectively reduced blood loss, transfusion requirements, and post-operative complications, supporting its use as a standard adjunctive therapy. These findings emphasize the importance of TXA in optimizing patient outcomes and minimizing surgical complications in TKR patients. Limitations of the study include the relatively small sample size and the exclusion of patients with certain comorbidities, which may limit the generalizability of the findings. Further research is warranted to validate these findings in larger patient cohorts and explore long-term effects.

## Introduction

For patients who suffer from severe knee arthritis and other degenerative joint diseases, total knee replacement (TKR) surgery is a life-changing intervention that can bring a significant transformation in your quality of live. However, its most obvious disadvantage is the loss of blood, which has surpassed 1,000 mL in some cases. On average, patients may require one to three units of packed red blood cells (PRBCs) to be transfused post-surgery [1]. The phenomenon has often been observed, leaving 18% to 67% of patients needing to have blood transfused [1], depending on factors such as patient comorbidities, the surgical technique used, and perioperative management. Not only do blood transfusions carry risks inherent in using blood products, but they also raise the chance of post-operative complications, such as prolonged hospital stays, infection, and even death [2].

Pre-operative and intraoperative management needs to be carefully planned over the TXA course [3]. One commonly used strategy in intraoperative prevention is a tourniquet, where blood flow is temporarily blocked to the surgical site. Though effective in cutting intraoperative blood loss, tourniquet use has created complications of its own, such as surgically induced trauma and fibrinolysis, in which the body starts to break up blood clots [3]. This may result in exaggerated blood loss.

In this context, an anti-fibrinolytic drug called tranexamic acid (TXA) has gained attention [4]. By blocking the breakdown of clots, TXA keeps them stable and reduces blood loss during and after surgery. Its role in TKR is particularly important, as it not only helps reduce blood loss but also gives some protection against post-operative thrombotic complications such as deep vein thrombosis [5]. TXA’s pharmacokinetics, with a biological half-life of about 2 hours in plasma and approximately 3 hours in synovial fluid, make it well-suited for maintaining hemostasis during critical surgical periods [5].

Despite the well-established benefits of TXA in reducing blood loss in TKR, there is a knowledge gap regarding its efficacy and safety specifically in elderly patients undergoing this procedure. The elderly population is at higher risk of both excessive bleeding and thrombotic complications due to age-related physiological changes and the higher prevalence of comorbidities [6]. Therefore, understanding the role of TXA in this particular patient group is critical for optimizing surgical outcomes and minimizing post-operative complications. This study aims to explore the efficacy of TXA in reducing blood loss when used in conjunction with a tourniquet during TKR in elderly patients, thereby addressing this gap in knowledge.

## Materials and methods

Study design and setting

This study was designed as a prospective descriptive-analytical, hospital-based randomized trial conducted across multiple hospitals in the Eastern Province of Saudi Arabia. The research spanned over eight months, from April 2023 to December 2023. The study was focused on evaluating the efficacy of TXA in controlling blood loss during TKR surgeries among elderly patients suffering from primary arthritis.

Study population

The study population consisted of patients diagnosed with primary arthritis who underwent TKR during the study period. A total of 79 patients, representing a diverse demographic in terms of age, gender, and nationality, were enrolled in the study. These participants were selected based on specific inclusion and exclusion criteria to ensure the reliability and validity of the study outcomes. Inclusion criteria included all patients scheduled for TKR surgery, who received TXA as part of their surgical protocol with no restrictions of nationality, age, ethnicity, gender, or socioeconomic. Patients with uncontrolled chronic diseases, particularly diabetes or hypertension, those with a history of thromboembolic disorders or hypersensitivity to TXA, and those who refused to provide consent for participation in the study were excluded.

Participant recruitment and consent

Participants were recruited through a block randomization process from the surgical schedules of the participating hospitals. Prior to inclusion, each participant was provided with detailed information about the study objectives, procedures, potential risks, and benefits. Written informed consent was obtained from all participants, allowing the use of their medical data for research purposes while ensuring the anonymity and confidentiality of their personal information.

Data collection

Data collection was meticulously planned and executed to ensure both accuracy and completeness. The collected data were categorized into several key areas: demographic data, including age and gender; surgical and clinical data detailing the TKR procedure, such as surgery duration, tourniquet use, dosage, and timing of TXA administration; outcome measures, including blood loss during surgery, the need for blood transfusions, incidence of post-operative complications such as deep vein thrombosis, and length of hospital stay; and complication rates, which recorded the frequency and type of any post-operative complications, including thromboembolic events and wound infections. Data were gathered using standardized forms, and, where feasible, data entry was verified by a second researcher to minimize errors.

Data management and statistical analysis

All collected data were entered into SPSS Version 26 (IBM Corp., Armonk, NY) for statistical analysis. The data were stored securely, with all personal identifiers removed to maintain patient confidentiality. Descriptive statistics, including mean, median, standard deviation, and frequency distributions, were calculated for the demographic and clinical variables. The sample size of 79 participants was determined based on a power calculation performed prior to the study, ensuring 95% power to detect a significant difference in the primary outcome measure, which was intraoperative blood loss.

Ethical considerations

Ethical clearance was obtained from the Ethical Committee of the Medical College at King Faisal University prior to the commencement of the study. The study adhered to the ethical principles outlined in the Declaration of Helsinki. Participant confidentiality was rigorously maintained, with all data anonymized and securely stored. The study posed no significant risks to participants, as the administration of TXA is a well-established practice in TKR surgeries. The study was self-funded, with discussions held with King Faisal University regarding potential additional funding and collaboration.

## Results

In the study, all participants received intravenous TXA at a dose of 1 g both before and after the surgery. The pre-operative dose was administered approximately 30 minutes prior to the surgical incision. The demographic analysis revealed a mean age of 66.11 years (SD = 6.32), with participants’ age ranging from 53 to 80 years. The gender distribution showed that 20 (25.3%) were male, while 59 (74.7%) were female. The majority of participants were obese (61, 77.2%), followed by overweight (15, 19.0%) and normal weight (3, 3.8%), based on BMI categories (Table 1).

**Table 1:** Demographic factors of the participants Data are presented as mean (SD) or number (percentage)

		N	%
Age	Mean (SD)	66.11 (6.32)	
	Min-max	53-80	
Gender	Male	20	25.3%
	Female	59	74.7%
BMI	Mean (SD)	33.8 (6.69)	
	Normal weight	3	3.8%
	Overweight	15	19.0%
	Obese	61	77.2%

**Figure 1:**
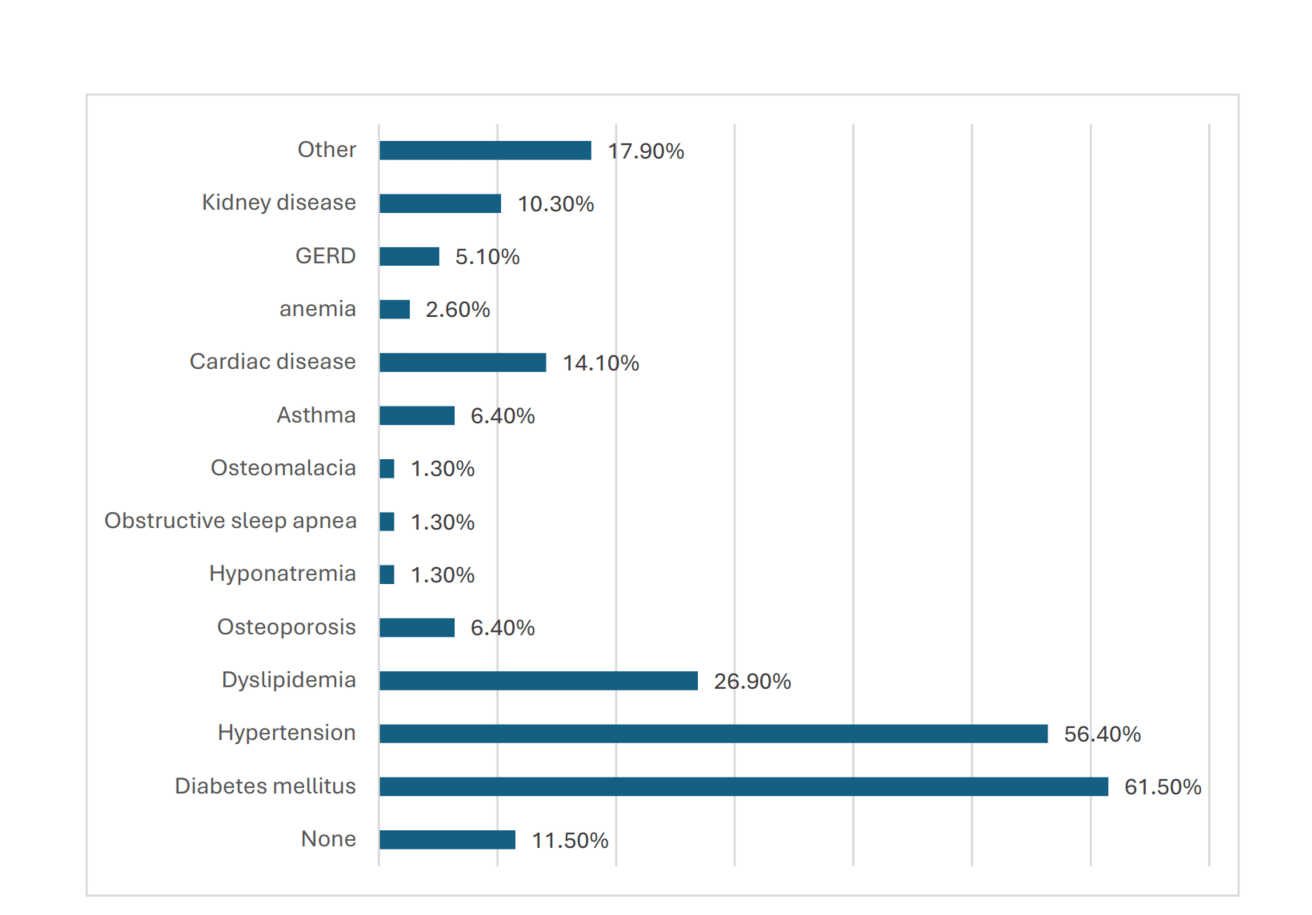
Comorbidities among the patients GERD, gastroesophageal reflux disease

Regarding comorbidities, the most prevalent were diabetes mellitus (49, 61.5%) and hypertension (45, 56.4%). Other comorbidities included dyslipidemia (21, 26.9%), cardiac disease (11, 14.1%), and kidney disease (8, 10.3%), among others. The patients' outcomes after surgery predominantly involved primary procedures (78, 98.7%), with only a small percentage undergoing revision (1, 1.3%) (Figure 1).

Pre-operative hemoglobin levels had a mean of 12.72 g/dL (SD = 1.48), which decreased post-operatively to a mean of 11.16 g/dL (SD = 1.53), resulting in a mean drop of -1.55 g/dL (SD = 0.94). The average length of hospital stay was 4.84 days (SD = 1.04). Nearly all patients (78, 98.7%) did not require PRBC transfusions post-operatively, and the majority (78, 98.7%) did not experience any post-operative complications. Only one (1.3%) patient developed melena as a post-operative complication (Table 2).

**Table 2:** Patients’ outcome after the surgery PRBC, packed red blood cell

		N	%
Type of procedure	Primary	78	98.7%
	Revision	1	1.3%
Hemoglobin level pre-operative	Mean (SD)	12.72 (1.48)	
Hemoglobin level post-operative	Mean (SD)	11.16 (1.53)	
Drop in hemoglobin post-operative	Mean (SD)	-1.55 (0.94)	
Length of hospital stay (days)	Mean (SD)	4.84 (1.04)	
Number of received PRBCs	0	78	98.7%
	1	1	1.3%
Post-operative complication	None	78	98.7%
	Melena	1	1.3%

## Discussion

The administration of TXA in TKR surgery has been a subject of considerable interest due to its potential to reduce blood loss and transfusion requirements [7]. In this study, all patients received TXA both before and after surgery, with the aim to assess its efficacy and safety. One of the key findings was the mean drop in hemoglobin levels post-operatively, which is a crucial indicator of blood loss during surgery. Comparing this result with existing literature on TXA use in TKR surgeries and those without TXA administration provides valuable insights into the efficacy of TXA in reducing blood loss and its impact on patient outcomes.

In the literature, TXA was shown to reduce blood loss and the need for transfusions without significantly increasing the risk of death, stroke, heart attack, or kidney failure [8,9]. In the field of orthopedics, TXA has been increasingly preferred because of its effectiveness and convenience, whether used intravenously or topically. The affordability, absorption rate, effectiveness, and minimal risks have contributed to the growing popularity of TXA in TKR [10,11]. The mean drop in hemoglobin levels post-operatively in our study was -1.55 g/dL, which aligns with findings from previous studies investigating TXA use in TKR surgeries. In a previous study, the authors found that the mean drop in the post-operative hemoglobin concentration in patients receiving TXA in TKR surgeries versus those who did not receive TXA was 0.6 g/dL (24 hours) and 1.3 g/dL (72 hours) compared to 1.5 g/dL (24 hours) and 2.3 g/dL (72 hours), with a mean drain collection of 474 ± 30.7 mL (24 hours) and 453.3 ± 37.7 mL (72 hours) [7]. Moreover, Gautam et al. reported that the total blood loss was lower in the TXA group than in the placebo group (427.6 mL vs. 911.6 mL; p<0.001) [12]. A review of nine randomized control studies showed that using TXA for patients having TKR greatly lowers the number of patients needing blood transfusion [13]. Additional research also found a decrease in the percentage of patients getting transfusions with this therapy [14]. Lozano et al.'s study showed that only 17.6% of patients on TXA needed red blood cell transfusion, compared to 54% in the control group during TKR [15]. Similar results were reported by Alvarez et al, who administered the same bolus amount. Studies raised doubts about the effectiveness of post-operative reinfusion drains and autologous transfusion in reducing blood loss and the need for transfusion after the use of TXA [16]. The same results were reported by many previous studies and indicated that TXA application in patients who underwent TKR is associated with a lower need for blood infusion and a lower drop in hemoglobin level compared to placebo or other strategies [17-19]. The substantial difference in hemoglobin drop underscores the importance of TXA administration in enhancing patient outcomes by minimizing the need for blood transfusions and associated complications.

In addition to assessing blood loss, our study evaluated various patient outcomes, including length of hospital stay, transfusion requirements, and post-operative complications. The results indicated a relatively short length of hospital stay (mean = 4.84 days) and low transfusion requirements, with the majority of patients not requiring PRBC transfusions post-operatively. These findings are consistent with previous studies demonstrating the positive impact of TXA on reducing transfusion rates and promoting early mobilization and discharge following TKR surgery [1,20,21].

Moreover, the low incidence of post-operative complications in our study population further supports the safety profile of TXA in TKR surgeries. Only one (1.3%) patient experienced melena as a post-operative complication, suggesting that TXA administration was well-tolerated overall. This aligns with the findings of meta-analyses and systematic reviews, which have consistently demonstrated the safety of TXA in orthopedic surgeries, including TKR [22-25]. Comorbidities such as cardiovascular disease, obesity, or renal dysfunction could potentially exacerbate post-surgical complications, including bleeding, thromboembolism, or infection [26]. While our findings suggest that TXA is effective and safe, future studies should investigate how pre-existing comorbidities influence the effectiveness of TXA and the incidence of post-operative complications. This would provide a clearer understanding of the broader applicability of TXA in diverse patient populations undergoing TKR.

It is essential to acknowledge the limitations of our study, including its single-center design and relatively small sample size. Another significant limitation is the absence of a control group, which restricts our ability to directly compare the efficacy of TXA with other interventions or with no TXA administration. The lack of a control group means that we cannot definitively attribute the observed reductions in blood loss and transfusion requirements solely to TXA, as other factors, such as surgical technique, patient comorbidities, or perioperative management, may have also played a role. The inclusion of a control group, such as patients who underwent TKR without TXA, would have strengthened the study by providing a baseline for comparison. However, due to ethical concerns about withholding a potentially beneficial treatment like TXA in a high-risk surgical population, we opted for a study design that ensured all patients received TXA. Future multicenter studies with larger cohorts are warranted to validate our findings and provide further insights into the effectiveness of TXA in TKR surgeries across diverse patient populations. Additionally, long-term follow-up assessments are needed to evaluate the impact of TXA on clinical outcomes such as thromboembolic events and implant survival.

## Conclusions

In conclusion, our study suggests that TXA administration in TKR surgeries may help reduce blood loss, as indicated by the modest drop in hemoglobin levels post-operatively. These findings support the widespread adoption of TXA as a standard adjunctive therapy in TKR surgeries to minimize blood loss, transfusion requirements, and associated complications. Further research is needed to optimize TXA dosing regimens and elucidate its long-term effects on patient outcomes in orthopedic surgery.
